# Cellular Immunotherapy and the Lung

**DOI:** 10.3390/vaccines9091018

**Published:** 2021-09-13

**Authors:** Sorcha Daly, Andrew O’Sullivan, Ronan MacLoughlin

**Affiliations:** 1 College of Medicine, Nursing & Health Sciences, National University of Ireland, H91 TK33 Galway, Ireland; s.daly37@nuigalway.ie; 2 Research and Development, Science and Emerging Technologies, Aerogen Limited, Galway Business Park, H91 HE94 Galway, Ireland; aosullivan@aerogen.com; 3 School of Pharmacy and Pharmaceutical Sciences, Trinity College, D02 PN40 Dublin, Ireland; 4 School of Pharmacy & Biomolecular Sciences, Royal College of Surgeons in Ireland, D02 YN77 Dublin, Ireland

**Keywords:** cellular immunotherapy, inhalation, respiratory disease, respiratory viruses, antibody, NK cell, DC vaccine, T cell

## Abstract

The new era of cellular immunotherapies has provided state-of-the-art and efficient strategies for the prevention and treatment of cancer and infectious diseases. Cellular immunotherapies are at the forefront of innovative medical care, including adoptive T cell therapies, cancer vaccines, NK cell therapies, and immune checkpoint inhibitors. The focus of this review is on cellular immunotherapies and their application in the lung, as respiratory diseases remain one of the main causes of death worldwide. The ongoing global pandemic has shed a new light on respiratory viruses, with a key area of concern being how to combat and control their infections. The focus of cellular immunotherapies has largely been on treating cancer and has had major successes in the past few years. However, recent preclinical and clinical studies using these immunotherapies for respiratory viral infections demonstrate promising potential. Therefore, in this review we explore the use of multiple cellular immunotherapies in treating viral respiratory infections, along with investigating several routes of administration with an emphasis on inhaled immunotherapies.

## 1. Introduction

Immunotherapy treatments pave the way for new personalised medicines through harnessing the patient’s own immune system, providing a promising cure for critical diseases [1]. More specifically, cellular immunotherapies (CIs) involve administering living cells to patients and have largely evolved since Sipuleucal T (Provenge), the first CI approved by the FDA in 2010. Since then, two additional CIs have been approved by the FDA, including tisagenlecleucel (Kymriah) and axicabtagene ciloleucal (Yescarta) [2]. Here, we discuss the published literature around this topic and provide insight into CIs and their application in the lung. The review methodology makes use of computerised searches of online databases in order to identify key publications. Databases include, but are not limited to: Scopus (Elsevier, Amsterdam, The Netherlands), ScienceDirect, Web of Science (Thomson Reuters, Toronto, ON, Canada), BioMed Central (Springer, Berlin/Heidelberg, Germany), and PubMed. Searches were also undertaken using Google Scholar. Online searches used relevant terms associated with elements of cellular immunotherapy and the lung, with combinations of the following terms: Cellular Immunotherapy, Inhalation, Respiratory Disease, and Respiratory Viruses. Citations in relevant publications were checked (backward citation searches) and papers citing relevant publications were studied (forward citation searches).

There has been significant development in CIs due to recent advances in immunology, molecular biology, and virology, along with technological breakthroughs in cell manufacturing and genetic engineering. CIs, which were once studied in research institutions on a small-scale, have now expanded to a larger scale in global commercial organisations [3]. Presently, a variety of CIs exist, such as Adoptive T Cell therapy (ATC), Dendritic Cell (DC) vaccine therapy, and Natural Killer (NK) cell therapy. Considerable efforts are being made to increase the efficiency of these technologies to ensure they are clinically applicable treatments [4].

Mucosal surfaces are the largest organs that protect our internal body surfaces from being exposed to the outside environment. They also prohibit pathogens and macromolecules from reaching the internal surfaces of the body when their entry is unfavourable [5,6]. The mucosal surfaces of the lungs and upper respiratory tract are frequently susceptible to infection [7]. These mucosal surfaces use a system of biological barriers with the aim of providing the body with protection from unwanted pathogens and external irritants. The mucosal barrier functions as an immunological organ and is well equipped to recognise and destroy any adverse exposures. Epithelial cells and numerous immune cells in the respiratory tract such as alveolar macrophages express pathogen recognition receptors (PRR) to facilitate robust immune responses, such as during an infection, which results in immune cell recruitment and inflammation. PRR also play an important role in recognizing molecules released by damaged cells known as damage associated molecular patterns (DAMPs) [8].

The physical mucus layer covering the mucosal epithelium, mucosal immune cells, lymphoid organs and underlying blood and lymphatic vasculature, along with the local immune system, act as a barrier against pathogens. However, these aforementioned barriers present an issue for CIs which must first pass through the mucus and epithelium before arriving at their localised therapeutic target [6]. It is well known that the induction of mucosal immunity via vaccination is a challenging task, and it often requires specific delivery systems and adjuvants for optimal efficacy [9]. This is also the case with CIs, as immune checkpoint inhibitors (ICIs) can be divided into two groups based on their infusion reactions. The European Society for Medical Oncology (ESMO, Lugano Switzerland) groups ICIs into either immune-related adverse events (irAE) or adverse events of special interests (AEoSI), with the goal of managing immunotherapy associated toxicities. Clinical data states that ICI use in several tumours is linked to an increased risk of irAEs in patients [10]. Pneumonitis is the most common irAE of the respiratory tract and frequently results in the halt of immunotherapy [11].

Immunotherapies can be subdivided into ‘passive’ and ‘active’ on the basis of their ability to employ the host immune system to fight diseases, such as cancer [12]. Typically, passive immunotherapy temporarily provides the body with exogenously produced immunological effectors in the manner of either target-specific antibodies or lymphocytes functionalised with target specific receptors [13]. Tumour-targeting monoclonal antibodies (mAbs) and ATC are immunotherapies sub-grouped into passive immunotherapies. Active immunotherapies, such as cancer vaccines and checkpoint inhibitors [12], become therapeutically active upon host immune system engagement only.

ATC is a type of CI that is considered to be a highly personalised treatment for cancer and involves the use of immune cells with anticancer activity. ATC is proposed to be a ‘living’ treatment as the cells administered can proliferate in vivo and preserve their antitumour effector role. ATCs can be used in numerous ways for the treatment of cancer, including tumour infiltrating (TIL), the genetic modification of T cell receptors (TCR) or modifying the T cells to express the chimeric antigen receptor (CAR). Using these approaches has a drastic effect on numerous cancer types, reducing the regression in melanoma, cervical cancer, lymphoma and leukemia [14]. An alternative use for ATC is in the treatment of viral infections. Multiple groups have expanded virus-specific T cells (VST) for the treatment of one particular virus or several viruses to eradicate active viral infections or, alternatively, regenerate antiviral immunity following a stem cell transplantation (SCT) [15].

Therapeutic cancer vaccines are classified as being part of the immunotherapy category. They are typically administered after a cancer emerges or has been removed, or alternatively can be used as a direct method in eliciting an immune response against a non-resectable tumour. The vaccines can be specific for tumour-specific antigens or tumour-associated antigens. Peptide, DNA, and mRNA vaccines are typically aimed at a single antigen or multiple antigens, with whole-based vaccines consistently aimed at multiple antigens [16]. DC-based vaccines are a form of CI and unlike other therapies they boost the patient’s own immune response towards their own tumour and present a low threat of toxicity [17]. Additionally, vaccines play a fundamental role in the control of emerging zoonotic viruses that have become a concerning global threat in recent times. Since 2000, zoonotic viruses have been the cause of several epidemics and pandemics, including, but not limited to, swine flu, Zika, Chikungunya, Ebola and more recently, COVID-19. Adaptive immune responses for protection are the main way in which virus-induced immunity is achieved prior to a virus infection. Advances in immunology and virology have led to a better understanding of molecular and cellular mechanisms of vaccine stimulation of immune responses. The success of viral vaccines is apparent in the worldwide eradication of smallpox virus and rinderpest [18].

NK cells are immune cells that recognise targets that are stressed by malignant transformation or infection. NK cells are advantageous in clinical application due to their large longevity and memory functions of past exposures. NK cell therapies are a type of CI that can be used to treat cancer or promote the benefits of a hematopoietic SCT. This is due to their lysis ability in tumours and their abnormal MHC Class I expression, along with their capability to produce chemokines and cytokines upon activation [19]. NK cells are also fundamental in the antiviral immune response, with patients having a hereditary NK cell deficiency shown to have increased susceptibility to viral infections. NK cells have a variety of techniques to eliminate virus-infected cells such as the engagement of extracellular death receptors and exocytosis of cytolytic granules [20]. CIs using NK cells have been successful in the treatment of hepatocellular carcinoma (HCC) caused by hepatitis B virus (HBV) and hepatitis C virus (HCV) [21]. Interestingly, there has been promising research for their applicability in treating severe acute respiratory syndrome coronavirus-2 (SARS-CoV-2), as they provide a protective and pathological role in this viral infection [22].

## 2. Application in the Lung

CIs to treat cancer, known as cancer immunotherapy, have been hugely successful in enhancing antitumour responses, resulting in less off-target effects than myeloablative treatments such as chemotherapy and other therapies that directly kill cancer cells [23]. CIs have proven to be a successful treatment for lung cancer, specifically small-cell lung cancer (SCLC) and can be achieved through ICIs, tumour vaccines and antigenic targets [24]. However, in light of the ongoing global pandemic, the focus of this review is the application of CI in treating viral respiratory infections. Respiratory viruses can have a huge impact on the health of both children and adults. An evolving societal challenge exists due to the widespread prevalence, a lack of sterilizing immunity, high morbidity, and lethality rates of diseases as a result of influenza, respiratory syncytial virus (RSV), coronaviruses, and rhinoviruses [25]. These respiratory diseases are the third leading cause of death globally and mortality rates are anticipated to carry on increasing in the forthcoming years. In the European Union (EU) the total cost of respiratory diseases is over EUR 380 billion, a significant economic burden which will increase in the coming years [26]. The high cost of CIs poses multiple challenges related to accessibility to patients, with the staggering cost of CAR T cell therapies ranging from EUR 373,000 to EUR 475,000 per dose. Assessing these obstacles and establishing practical solutions is hugely important as the field continues to evolve. A decrease in the price of CIs would allow for the potential to broaden their use in treating not only cancer, but also respiratory lung diseases [27].

## 3. Respiratory Diseases

### 3.1. ARDs

Acute respiratory distress syndrome (ARDs) is caused by non-cardiogenic pulmonary adema and manifests as a disorder of acute respiratory failure [28]. The current global pandemic has resulted in an urgency to understand the pathogenesis of SARS-CoV-2-induced lung injury and establish effective therapies. This is particularly relevant in the case of ARDs as clinical data reveals that severe COVID-19 most commonly results in viral pneumonia-induced ARDs [29]. Approximately 5% of patients infected by SARS-CoV-2 require intensive care as a result of ARDs, with fatality rates varying from 30–60% [30,31].

### 3.2. RSV

RSV is a member of the paramyxoviridae family that accounts for the leading cause of bronchiolitis and respiratory tract infections necessitating hospitalisation [32]. A study by Shi et al. recorded that RSV resulted in 3.2 million hospitalisations, 59,600 deaths of children under the age of five, and 118,200 deaths amid all age groups, per year [32,33]. Infants that fall into high-risk groups have a greater chance of acquiring severe RSV, such as underlying medical conditions including chronic lung disease of prematurity (CLDP), bronchopulmonary dysplasia (BPD), immunocompromised infants or children suffering from severe neuromuscular illnesses. An apparent link also exists between severe RSV infection and wheezing throughout early life, along with asthma and overactive airways in later life [34]. RSV usually multiplies in the epithelial lining of the nasopharynx and upper respiratory tract but is capable of proliferating towards the small bronchioles or alveoli of the lower respiratory tract. The surface of the virion consists of attachment (G) and fusion (F) glycoproteins that have fundamental roles in entry [35]. The treatment of RSV is undoubtedly restricted by the paucity of effective antiviral agents, aside from ribavirin. The mAb palivizumab is presently the only intervention licensed for the prophylaxis of severe RSV. However, its use is reserved for subsets of high-risk infants including those born preterm and those suffering from CLDP, BPD, or cogenital heart disease (CHD). A large number of RSV cases account for children without any underlying health conditions, highlighting the requirement for an increase in alternative, cost-effective treatments for RSV [36]. Another major concern is the susceptibility of reinfection with RSV, as the neutralizing antibodies produced by the primary RSV infection are only partially protective for a very limited amount of time [37]. CI could be considered as a potential future treatment strategy for treating respiratory viruses.

### 3.3. SARS-CoV-2

Coronavirus 2019 (COVID-19) occurs as a result of the virus, SARS-CoV-2 [38]. COVID-19 was first identified on 31st December 2019 in the city of Wuhan in China. Patients displayed clinical symptoms of dry cough, dyspnea, fever and bilateral lung infiltrates on imaging. On 30th January 2020, the WHO declared the COVID-19 outbreak in China to be a Public Health Emergency of International Concern presenting high risk to countries, particularly those with weak health systems [39]. On 11 March 2020, the WHO subsequently declared the COVID-19 outbreak a global pandemic, prompting an acceleration in emergency response procedures worldwide [40]. The SARS-CoV-2 virus primarily affects the lungs, however other organs and systems can be affected. Patients infected with this virus can have symptoms ranging from mild or severe respiratory failure up to multiple organ failures [41]. SARS-CoV-2 infects human cells through binding to the angiotensin-converting enzyme-2 (ACE2), which is largely expressed on epithelial cells of the respiratory tract. Attachment to ACE2 is enabled by the viral spike (S) glycoprotein, extruding from the surface of the virus and also through cleavage of the S protein by host cell proteases. Inflammation is a mediator of the secondary effects, and the immune response known as a ‘cytokine storm’ often plays a role in COVID-19 [42].

### 3.4. Additional Respiratory Viruses

Respiratory viruses are a common cause of disease in humans, having a global impact on both morbidity and mortality [43]. The current global pandemic has put a huge emphasis on respiratory viruses and their potential influence on society. A study by Burrel et al. investigated the degree of co-infections with SARS-CoV-2 with other respiratory viruses, highlighting the increased interest in the other major viruses infecting the respiratory tract [44]. Aside from coronavirus, the most prevalent respiratory viruses that are transmissible worldwide include the influenza virus, RSV, parainfluenza virus, metapneumovirus, rhinovirus, adenovirus and bocavirus [43]. There have been multiple vaccines and antiviral drugs on the market to treat respiratory viruses such as oseltamivir for influenza virus, palivizumab to prevent RSV infection, and various COVID-19 vaccines such as Pfizer-BioNTech [45,46,47]. Although these treatments are proven to have clinical benefits, there are very few that have spectrums of activity comparable to the antibacterial agents, penicillin or sulfa [48]. Additionally, a major concern is that it is not always gauranteed that infection with RNA viruses will lead to long-lasting protection against reinfection [49]. A shortage of effective vaccines and antiviral drugs is evident, while CIs could be considered a new feasible option to enhance the efficacy of treatments. 

## 4. Cellular Immunotherapies

### 4.1. Monoclonal Antibodies against COVID-19

There is a pressing need to examine novel therapies to treat COVID-19 in order to decrease mortality rates, virus transmission and diminish any other future outbreaks. Immunotherapy serves as an obvious approach as it is highly effective in the treatment of infectious diseases. A type of immunotherapy called mAbs have gained much attention in the pharmaceutical industry in recent times [50]. An mAb is any antibody which has novel specificity and is derived from a single-cell B clone [51]. These therapeutic antibodies are primarily produced in mammalian host cell lines such as the NS0 murine myeloma cells, PER.C6^®^ human cells and Chinese hamster ovary (CHO) cells. CHO cells exist as the primary host for generating such therapeutic proteins [52]. In the case of mAbs, the cells trigger the therapy, classifying them as cell-derived therapies more so than cellular therapies. Cell-derived therapies refer to the secreted factors or products produced by the cells such as extracellular vesicles (EVs) or the conditioned medium (CM). The mAbs are more likely to fall into this cell-derived category, as cellular therapies refer to when the cell itself is the therapy [53,54].

The mAbs can bind to one specific substance in the body, and in the case of SARS-CoV-2 they identify the S1 fragment, with some recognizing the S2 fragment and other mechanisms functioning in neutralisation. The combination of mAbs targeting S fragments in the virus has been successful in the detection of multiple epitopes and in vivo cells that can be efficacious at the viral level. Passive antibody therapy is a viable means to limit COVID-19 progression. However, this therapy should be used with caution as there have been some responses which display inflammation leading to acute lung fatal injury, with some SARs patients not surviving [55].

The mAbs against IL-6 receptors such as tocilizumab (TCZ) and sarilumab are used in the treatment of rheumatoid arthritis and have been approved by the FDA for controlling cytokine release syndrome (CRS) manifesting in excessive cytokine production and rapid multiorgan damage. As cytokine storms are involved in COVID-19 onset, they are hypothesised to employ suppressive effects on inflammation in patients infected with SARS-CoV-2. Three clinical trials for TCZ have been approved for COVID-19. Bevacizumab may be considered as it is a monoclonal anti-vascular endothelial growth factor (VEGF) antibody that competes with VEGF receptors on the surface of endothelial cells for VEGF binding. VEGF is a potent vascular permeability binder and is generated by multiple inflammatory and epithelial cells [56]. Studies have revealed that there is a striking increase in plasma VEGF levels in patients with ARDs [57]. This type of mAb is considered to be a possible therapeutic approach for ARDS, which is a prevalent complication of COVID-19. Further highlighting its clinical application in the treatment of COVID-19 [56].

A recent study carried out by Nieto et al. generated an alpaca Nanobody with potential future therapeutic application due to its high specificity for the receptor binding domain (RBD) of the SARS-CoV-2 spike protein. The group were able to design a single-step selection of Nanobodies through the use of a simple density gradient centrifugation of a bacterial library. Nanobodies could be isolated using a rapid approach, entailing an optimised immunisation regimen incorporated with a VHH *E. coli* surface display [58]. When a VHH domain is isolated, it is known as a Nanobody or a single domain antibody (sdAb). Nanobodies have high clinical potential as they can be employed as therapeutic bullets against pathogens, tumours, or even chronic diseases [22,59,60]. They are favourable over classical antibodies as they can be effectively generated in prokaryotic expression systems. Nieto et al. managed to provide an approach to successfully speed up the identification of Nanobodies which in turn allows the formation of diagnostic and therapeutic means against COVID-19, along with other viruses and diseases [58].

### 4.2. NK Cell Therapy against COVID-19

Patients with mild and severe COVID-19 displayed a decrease in NK cell circulation and function in comparison to healthy individuals. NK cell products are typically expanded in vitro in the presence of cytokines or via co-culture with target cells prior to infusion into patients. Moreover, there is an inverse correlation between reduced NK cell levels and disease severity. As seen in Table 1, an allogeneic, off-the-shelf, cryopreserved NK CI was produced by Celularity as the first investigational drug to be approved by the FDA for clinical testing in patients suffering from COVID-19 [59]. Another avenue for the treatment of COVID-19 is the investigation of genetically modified NK cells. CAR-NK cells are engineered to express almost any receptor of interest and were initially created to amplify the ability of NK to destroy cancer cells through receptors targeting EGFR or CD19. The use of CAR-NK cell therapy in the management of viral infections still requires more testing in large-scale clinical trials; however, its promising outcomes in immunocompromised cancer patients suggests it may be successful in COVID-19 patients [22].

There are a lack of studies investigating the role of NK cells in COVID-19 pathophysiology, as well as in other coronaviruses [22]. A study by Zheng et al. assessed the NK cell function and phenotype using peripheral blood from COVID-19 patients. Upon administration, it appeared that there was an inverse correlation between NK cell levels in the peripheral blood with disease severity. Additionally, COVID-19 patients suffering with a severe case of disease had notably lower circulating NK cells in comparison to patients with milder cases. There was also an increased expression of the inhibitory receptor NKG2A in NK cells circulating in severe disease. Moreover, the cells had a hyperresponsive phenotype with decreased levels of IFN-γ, tumour necrosis factor (TNF)-α, IL-2, and granzyme B, despite degranulation being maintained. Patients recovering from COVID-19 had higher NK cell numbers and lower NKG2A expression, in comparison to patients with the active disease [61].

Liao et al. carried out single-cell RNAseq on cells acquired from the bronchoalveolar lavage fluid of patients with both severe and mild COVID-19. They revealed that patients suffering with COVID-19 had remarkably more NK cell infiltrates in the lungs, although reduced numbers of NK cells were seen in severe COVID-19 cases. In addition, there were high levels of expression of the KLRC1 and KLRD1 genes by NK cells [60].

### 4.3. Immune Checkpoint Inhibitors against COVID-19

Immune checkpoints refer to various inhibitory pathways that are inherent to the immune system. They are fundamental for regulating the duration and extent of physiological immune responses in peripheral tissues to diminish collateral tissue damage. Tumours can effectively modulate checkpoint pathways as a mechanism of immune resistance. For this reason, ICIs act to revert this by regulating various immune responses [62]. Aside from their important immunosurveillance mechanism in cancer, checkpoint blockades can be used for treating human infectious diseases such as viruses. PD-1 target therapies have been proven to efficiently suppress tumour growth and decrease viral load. The ICI anti-PD1, Camrelizumab, is being investigated for the treatment of COVID-19 patients. It is important to understand that checkpoint inhibitors may cause harm by adding to the cytokine storm of COVID-19, however, in contrast, the recovery of immunocompetence may have an effective or therapeutic effects against viral infection. Further studies using checkpoint blockades for infectious diseases are required before they can be fully considered for viral infections [63].

The mAbs are usually tailored to prohibit virus entry through disrupting the interaction between host cells, the ACE2 receptor, and the S protein. However, the administration of ICIs is a viable alternative as there is a high complexity in immune cell count, regulatory protein expression, and cytokine secretion [64]. A randomised control clinical trial is currently assessing the efficiency of the anti-PD1 agent and thymosin on COVID-19 patients suffering with lymphopenia and severe respiratory failure. Phase II of this trial goes on to evaluate the consequences of anti-NKG2A (monalizumab), anti-C5aR (avadoralimab) and autophagy inhibitor GNS561 administration to COVID-19 patients with either advanced or metastatic cancer. This trial consists of four arms, divided into two cohorts of patients: one consists of patients with mild and asymptomatic symptoms; another consists of patients suffering from moderate or severe symptoms [64]. NKG2A and C5aR are ICIs that reside in a more unique class. T cell-expansion and anti-tumour immunity is amplified when they are inhibited [65,66].

### 4.4. Adoptive T Cell Therapy against COVID-19

ATC has been effectively used for viral infections for decades, making its application in SARS-CoV-2 a rational therapeutic approach. VSTs from an autologous or allogeneic source can be expanded in vitro and infused to restore effective antiviral immunity, successful in treating several viral infections [67]. SARS-CoV-2-specific T cells can be isolated from the circulation of recovering donors, and by using SARS-CoV-2 peptides they are expanded and utilised for treating severe COVID-19 cases. However, ATC treatment for COVID-19 does not come without its limitations, as there are some treatment-related toxicities associated with its use. Furthermore, genetic restrictions mean it is not feasible to use unmatched allogeneic T cells and the prolonged stimulation of in vitro expanded T cells to achieve necessary cell yields as they may demonstrate functional exhaustion. It is also possible that disease complications could be enhanced by transferred T cells adding to the cytokine storm. Clinical trials based on ATC for COVID-19 are still ongoing and utilise already-collected SARS-CoV-2-specific T cells isolated from recovered patients to treat COVID-19 patients with a high risk of respiratory failure [68].

A study carried out by Ferreras et al. evaluated the presence of SARS-CoV-2-specific T cells within CD45RA-memory T cells in convalescent donor’s blood. Memory T cells are known to mount a fast response to infection and supply immune protection that is long-lasting, dampening the severity of COVID-19 symptoms. Interestingly, CD45RA-memory T cells offer protection from additional pathogens experienced by patients in their lifetime. It is found that the SARS-CoV-2-specific T cells within the CD45RA-memory T cells from the blood can be readily, efficiently, and quickly isolated via CD45RA depletion. It is possible that these cells can be used in the clearance of virally infected cells and offer T cell immunity to ensure reinfections. The cells represent an off-the-shelf living drug that has huge potential in treating both moderate and severe cases of COVID-19 patients at risk of hospitalization [69].

### 4.5. Adoptive T Cell Therapy against Other Respiratory Viruses

Viral infections continue to be the leading source of morbidity and mortality following a hematopoietic SCT [70]. Respiratory viruses play a significant role in causing issues in patients post transplantation, including adenovirus, influenza, and RSV. Immunotherapy strategies for respiratory viruses can often be limiting, with influenza as an exception, as their viral immunology is poorly understood in comparison to viruses such as Epstein Barr Virus (EBV). As these viruses can cause morbidity during the early stages post transplantation, it means that it is much more difficult to generate cytotoxic T lymphocytes (CTLs) from bone marrow transplant (BMT) donors [71]. One study reported that donor leukocytes may be active in RSV. A patient with plasma cell leukemia who relapsed 1 year after transplantation and developed RSV-interstitial pneumonia was administered with donor lymphocytes. After one week of donor lymphocyte infusion (DLI) the patient’s respiratory status ameliorated and a nasal swab was negative for RSV. Unfortunately, the patient died 3 weeks after DLI from septic shock. There was no RSV present in the bronchoalveolar lavage fluid after death, suggesting the promising role for T-cell therapy in RSV treatment [72].

Adoptive immunotherapy using VSTs has also been used to treat immunocompromised patients suffering with severe viral diseases. The therapy can be employed to treat several viruses such as cytomegalovirus (CMV), EBV and the common respiratory virus, adenovirus [73]. Primary immunodeficiency disorders (PID) are an increasing group of hereditary aberrations of immunity, which vary in severity from mild disorders in late childhood or adulthood to severe forms in early infancy [74,75]. Herpesviruses such as CMV and EBVs, as well as respiratory viruses such as adenoviruses, are a prevalent cause of mortality prior to and during hematopoietic STCs [73]. Although there has been much progress in antiviral pharmacotherapy, some of these agents are associated with notable toxicities, and seldomly facilitate viral control without the restoration of T cell immunity [76,77]. A selection of antigen-specific T cells using major histocompatibility complex multimers, cytokine capture technologies, or ex vivo expansion preceding antigen stimulation, are the present methods of VST production [78,79,80]. A total of nine patients have received VST therapy for adenoviremia in clinical, published studies. Of those patients, four received VSTs derived from stem cell donors, while the remaining five received VSTs derived from third party donors. The clearance of adenoviral infection was observed in eight out of the nine patients, underpinning the effectiveness of the approach regardless of the particular strategy used. Adenovirus-specific VSTs were given to eight patients as prophylaxis and during the follow-up period of approximately one year, none developed adenoviral infection [73].

ATC therapies such as VSTs are anticipated to have a safe and effective impact on patients suffering from community-acquired respiratory viruses such as RSV. Vasileiou et al. described an approach that quickly produces a single preparation of polyclonal VST specific for 12 immunodominant antigens derived from target viruses such as RSV, influenza, parainfluenza virus (PIV), and hMPV under Good Manufacturing Practice (GMP) compliance. The expanded cells have a high specificity for viral targets and are safe for clinical use due to their ability to react and kill viral antigen-expressing cells. The multi-respiratory virus-targeted cells (multi-R-VST) aim to administer a broad-spectrum effect in treating immunocompromised patients suffering with respiratory infections [81].

### 4.6. CAR T/CAR-NK Cell Therapy

CAR T cell therapy falls under the umbrella term of ATC and is possibly the most successful clinical therapy in this group as it cures 25–50% of patients with B-cell malignancies who were once incurable [82]. CAR is a modular fusion protein consisting of an extracellular target binding domain which is derived from a single-chain variable fragment (scFv), a spacer domain, transmembrane domain, and an intracellular domain holding CD3z with costimulatory molecules [83]. The CAR T cells bind to target cell surface antigens via the scFv domain to mediate MHC-unrestricted cell killing [84]. CAR T cell therapy has had undoubtable success in hematological malignancies, heightening our interest in their applicability to treat respiratory viruses such as SARS-CoV-2. 

Human macrophages were engineered with CARs to exhibit anti-tumor potential and, as they are crucial effectors of the innate immune response, it was thought CAR macrophages could be utilised to fight SARS-CoV-2 in a study carried out by Fu et al. [85,86]. The limitation of this study resided in the hyperinflammatory macrophage response which could be extremely harmful to the host, specifically those with severe infections, such as SARS-CoV-2 or CRS. This accounts for the most notable complication linked with CAR T cell therapy. This study produced numerous CARs based on the recognition of the S protein and evaluated their efficacy to induce phagocytosis of SARS-CoV-2 virions. MER Proto-Oncogene Tyrosine Kinase (MERTK) is highly expressed on macrophages and has numerous ligands such as Gas6 and protein S. The study showed that one CAR with the MERTK intracellular domain did not display a significant killing effect in antigen-expressing cell-based models in comparison to other CARs but did exhibit the antigen-specific clearance of SARS-CoV-2 virions in vitro without the secretion of proinflammatory cytokines. This work showed that CAR macrophages could serve as promising therapeutics for COVID-19 and that the MERTK-based CAR receptors did not upregulate proinflammatory cytokines. Additionally, direct virion clearance was displayed by CAR macrophages [86].

As previously mentioned, another interesting and novel use of CAR technology is CAR-NK cells. In contrast to T cells, NK cells recognise the presence of self-MHC class I molecules and determine stress-induced ligands on tumour cells [82]. Ma et al. explored the potential of CAR-NK cell therapy in treating COVID-19 as it was yet to be fully understood. The author addressed the need for alternative treatments as certain subsets of people may not be responsive to vaccines. CAR-NK cells have an advantage over CAR T cells in that they do not have the additional risk of developing severe graft versus host disease (GVHD) and also have reduced host cytotoxicity levels. This study aimed to generate a unique approach for the production of CAR-NK cells for targeting SARS-CoV-2 employing the scFv domain of S309. They conveyed that this procedure would preserve activity against the Spike gene variants that are spreading around the world rapidly. It was also shown that S309-CAR-NK had superior cytotoxic abilities when compared to Spike-protein-targeting CR3022-CAR-NK cells. Using CAR-NK cells as an effective strategy for treating COVID-19 may be feasible; however, to elevate this research even further preclinical animal models should be used [87].

The versatility of CAR T cell therapy extends beyond the treatment of respiratory viruses as it can be used for immunodeficiency viruses including HIV, and numerous hematological cancers [88,89]. A study by Liu et al. confirms this as the group developed a broadly neutralizing antibody(nNAb)-derived CAR T cell therapy for the treatment of HIV-1. They discovered that the CAR T administration was safe and tolerable in all 14 of the participants and viral loads were significantly reduced. HIV-1 variants were analysed before and after CAR T administration and indicated that CAR T exerted pressure on rebound viruses, resulting in an array of viruses with less diversity of mutations against CAR T cell therapy [88]. This work highlights the adaptability of CAR T cell therapy and how its potential will continue to expand with an increase in supporting clinical trials.

### 4.7. DC Vaccines against COVID-19

SARS-CoV-2 infects respiratory DCs, along with lung type2 alveolar cells and epithelial cells. This may account for the robust immunopathology associated with COVID-19 infection [90]. A dendritic cell-specific intracellular adhesion molecule-grabbing nonintegrin (DC-SIGN) is a C-type lectin expressed on the surface of DCs located in peripheral mucosa and was observed as playing a fundamental role in the attachment of numerous viruses to host cells. Cells expressing DC-SIGN and DC-SIGNR appear to enhance infection with SARS-CoV-2 [91]. DC vaccines have proven to be effective in treating cancer through their anti-tumour immunity mechanisms, which are enhanced further when co-administered with other adjuvants or interventions. COVID-19 has an extremely harmful effect on the population as a whole, but particularly the more vulnerable, such as cancer patients. With the present understanding of the virus, cancer and DC nature, a therapeutic approach that could be considered is using one strategy to target both COVID-19 and cancer. This may involve tailoring a DC vaccine that could block viral infection and act as an immune-boosting approach, attributed to its effective antigen-presenting ability, which in turn sensitises the immune system to simultaneously act against the tumour and virus. To fully benefit from the complementary actions of DCs, multiple DC subsets may be used to create the vaccine [4].

### 4.8. Potential of Vaccines and CIs against Viral Shedding

Viral shedding is a hugely important factor to identify and limit when considering viral diseases, especially at the height of a global pandemic. Determining the viral shedding of SARS-CoV-2 helps to confirm the number of days an infected person should stay in isolation. It is found that patients with severe COVID-19 have a much higher viral load and a longer interval of shedding in comparison to mild cases [92]. There have been several studies proving that prolonged viral shedding can occur in SARS-CoV-2 patients and is an important consideration when discontinuing isolation [93]. A recently published study examined 41 cases of severe COVID-19, discovering the median duration of viral shedding to be 31 days [94,95]. It is well-known that many viral vaccines have the ability to reduce viral shedding, potentially lessening the spread and number of cases. One study assessed if the intranasal route of administration to deliver the adenovirus-based vaccine ChAdOx1 nCoV-19/AZD1222 decreased viral shedding. They found that viral loads in swabs from intranasally vaccinated hamsters were reduced when compared to the control hamsters, with no infectious virus detected in the lung tissue when placed in direct contact with infected hamsters. The group also carried out this experiment in rhesus macaques and found a decrease in viral concentration from nasal swabs, along with a reduced viral load in bronchoalveolar lavage and in the lower respiratory tract. This study proved that the intranasal vaccination of ChAdOx1 nCoV-19/AZD1222 reduced viral concentration in two separate animal models with SARS-CoV-2, thus decreasing viral shedding [96].

As vaccines are seen to be effective in reducing viral shedding, an obvious avenue to explore was the effect of CIs. A study by Goyal et al. investigated the effect of multiple therapeutics on the duration of SARS-CoV-2 shedding, including antivirals such as remdesivir, inhibitors such as selinexor, and CIs such as NK cell therapy. They predicted that NK cell therapy would decrease the lifespan of infected cells considerably. It was also concluded that CIs would have limited success if started before peak shedding. This study uses a mathematical model to project the potential of antiviral drugs and CIs in reducing viral shedding; however, in order to confirm these findings, preclinical and clinical trials must be carried out [97].

## 5. Routes of Administration

### 5.1. Novel Delivery Routes

Improving response rates to several types of immunotherapies is fundamental to enhancing efficacy and managing serious adverse effects. Novel methods for CI administration in a safer and controlled approach could potentially lessen certain toxicities and increase the capacity of the therapeutic agent. Improved delivery technologies may increase the concentration of immunotherapies within diseased tissues, allow for more efficacious targeting of the specific tumour or site of infection and decrease off-target effects [23]. There is continuous investigation into novel delivery platforms for immunotherapies such as nanoparticles, biomaterials, scaffolds, implants, and cell-based methods [98]. Nanoparticle-based therapeutic approaches have become increasingly popular as they can effectively overcome biological barriers, deliver hydrophobic therapies, and favorably target certain disease sites [99,100,101,102]. Nanocarriers can include different types of formulations including lipid-based, metal-based, polymeric and branched polymeric, and magnetic and mesoporous silica [103,104]. Nanoparticle-based therapeutics have had a massive impact on lung cancer, particularly in the diagnosis, screening and treating of primary and metastatic tumors [103,105,106]. In addition to this, nanoparticles have the ability to positively modify biodistribution, escalating the therapeutic efficacy and decreasing the nonspecific toxicity of powerful anticancer drugs. They are advantageous in that they have significant biocompatibility, can protect nucleic acids from degradation and deliver genes in vivo to cancer cells [103]. 

Implantable biomaterial scaffolds have the capacity to be pre-filled with immune agents, bioactive factors, or cells and implanted into a tissue space that is resected or subcutaneously through minor surgery. Immune cells can be placed into scaffolds and activated to allow a gradual release of immunoregulatory agents [107]. In addition to this, a bioartificial lung could be produced whereby the blood–gas exchange surface is produced from either synthetic or natural scaffolds that contains stem or progenitor cells to imitate effective tissue of the lung [108]. DC vaccines have shown to be successfully used to treat cancer such as lung cancer and respiratory viruses including COVID-19 [4,109]. However, the vaccines require the complicated modification of cells first in vitro, resulting in the majority of cells dying upon transplantation. To tackle this challenge, implantable biomaterials can be utilised, acting as a physical conformation to bring in and program DCs for immunotherapy in situ. The drug delivery device comprises of the polymeric scaffold, managing the delivery of bioactive compounds in space and time for the recruitment of DCs and their subsequent proliferation [23].

### 5.2. Intravenous Route

The intravenous (IV) route is typically the most favorable route as it has the ability to deliver large doses of medication to the body in a direct manner [110]. However, administering drugs via the IV route can often lead to the drug being diluted in the plasma [111]. The target is usually not located in the plasma or the blood, meaning the drug must travel to the target organ, such as the lung in respiratory diseases. Moreover, following systemic administration, the plasma time concentration is typically viewed as a surrogate time concentration profile for the specific organ and site within the organ [112,113]. There are no drug transporters or appropriate barriers that exist between the lung tissue and the blood which suggests that free pulmonary concentrations are not necessarily higher than in the plasma. This in turn implies that systemic administration is unable to attain pharmacokinetic (PK) selectivity [113].

### 5.3. Intratracheal Instillation

Intratracheal instillation is an approach that is applied to deliver multiple different agents including pathogens, toxins, and therapeutic agents. The intratracheal method often includes the instillation of the formulation, which is non-invasive or alternative to direct injection into the trachea [114]. Instillation has some advantages over inhalation, mainly that the delivered dose to the lungs can be guaranteed and that it is a simpler route with a shorter delivery time [115,116]. Despite these advantages over the inhalation method, some concerns still remain regarding the instillation technique; one of which is that the upper respiratory tract is diverted by intratracheal instillation, which is often a fundamental target site for an inhaled product. It is also important to note that the distribution of the drug may be impacted by the instilled vehicle within the lung and can potentially induce effects itself or change the outcomes of the drug on the lungs [115]. The intratracheal route has been successfully used for administering cell therapies for lung disorders such as chronic obstructive pulmonary disease [117]. This delivery method has not been tested for administering CIs to the lung but may be a viable option to consider in the future due to its non-invasive manner. 

### 5.4. Inhalation

The lung serves as an appealing target for therapeutic administration, contributing a broad amount of blood vessels for systemic delivery, whilst also enabling local delivery for lung diseases such as lung cancer or respiratory infections [118]. Inhalation exists as one of the oldest delivery methods to treat respiratory diseases [119]. When medication is inhaled it can be transferred directly into the airways, which are the target site for obstructive respiratory diseases such as asthma and COPD. This enables a faster onset of action to be achieved with the use of reduced drug concentration, eliciting less side effects in areas where the action is not required [120,121]. Aerosol delivery devices have advanced hugely in the past 60 years from the standard pressurised metered-dose inhaler to various types of inhalers and devices such as valved holder chambers, dry powder inhalers, and soft mist inhalers, including smart inhalers and nebulisers [122,123,124]. 

Nebulisers are among the most common devices used in lung-targeted delivery of high-value therapeutics in the acute and critical care area [110,125]. They have multiple advantages over alternative methods, such as metered dose-inhalers and dry powder inhalers which facilitate high levels of drug delivery (by mass) and the potential for minimizing the escape of both fugitive- and patient-derived emissions [126,127,128,129]. An external energy source is used to aid their active aerosolisation mechanism and eliminates the need for hand–breath coordination [118]. They are considered to be favourable over other delivery methods due to their use of aqueous formulations and their capacity to deliver process-sensitive proteins, peptides and biological medications [130]. The three main types of nebulisers are ultrasonic nebulisers, jet nebulisers, and mesh nebulisers. Jet nebulisers utilise gas flow from a central air supply or, alternatively, from a compressor. The liquid drug is atomised as the gas passes into the reservoir. Large and small droplets are contained within the atomised liquid and directed to a baffle. Ultrasonic nebulisers differ in that they use the vibration of a piezo electric crystal to produce a cascade of large and small droplets. Mesh nebulisers are divided into two groups: static mesh and vibrating mesh nebulisers. Static mesh nebulisers consist of an ultrasonic transducer in close proximity to the liquid to create vibration of the liquid drug and force the liquid through the static mesh. There are around 1000–6000 holes contained within the mesh with a diameter of 3–6 μm and the particles produced by movement through this mesh have the ability to be directly inhaled by the patient. Moreover, the vibrating mesh nebulisers cause mesh deformation or vibration through a piezo element in contact with the mesh [129,131,132].

### 5.5. Inhaled Monoclonal Antibodies

The scientific principle for using inhalation as the delivery method for mAbs is based on two main lines of evidence, the first being that only a small number of mAbs are observed in the lungs and bronchoalveolar lavage (BAL) after IV administration. Secondly, the majority of drugs used for the treatment of lung diseases, aside from omalizumab, work mostly in the lungs as opposed to the periphery. Therefore, the inhaled route serves as an evident route for amplifying the dose of mAB delivered to the target tissue with minimal side effects [131].

Maillet et al. conducted a study to determine the feasibility of using nebulisers as a delivery route for therapeutic antibodies. A chimeric IgG1-targeting the epidermal growth factor receptor (EGFR), known as Cetuximab, was nebulised using three different types of delivery systems such as a jet nebuliser, a mesh nebuliser, and an ultrasonic nebuliser. They measured the aerosol size distribution with a cascade impactor and aerosol droplets were viewed using optical microscopy. They found that the three nebulisers exhibited similar aerodynamical features through the aerosol particle clouds produced. However, the nebulisation using the jet and ultrasonic devices resulted in IgG aggregates in the liquid phase. The mesh and jet nebulisers were observed to preserve the binding affinity to EGFR and cetuximab’s inhibitory activities. This work confirms that inhalation is a promising delivery route for mAbs in the treatment of lung diseases such as cancer [133].

The mAbs are typically administrated intravenously for treating cancer; however, alternative routes may be favourable for respiratory diseases as the antibodies do not passively diffuse via the various compartments of the body. A recent study looked specifically at the PK of nebulised mAbs in non-human primates through continuous sampling in lung parenchyma using micro dialysis. PK results, along with pharmacodynamics (PD) and toxicity profiles of Abs, are crucial to obtain prior to clinical trials. It is also a significant area of investigation as PK results are particularly difficult to interpret with inhalation as a delivery route. In vivo micro dialysis is used as a means of PK sampling as it is a semi-invasive technique that quantifies unmodified drugs in the interstitial spaces of several tissues, including the lungs. Microdialysis proved to be a reliable means to understand and study the in situ behavior of inhaled mAbs targeting soluble antigens [134].

Another study looked at the fate of inhaled mAbs proceeding the deposition of aerosolised particles in the respiratory system. Their study model consisted of a cetuximab and anti-EGFR antibody to demonstrate that, after its delivery through airways, the mAb gathered rapidly in normal and cancerous tissue, at twice the concentration reached by IV injection. A mouse model with lung tumors expressing the desired antigen was utilised to investigate this. PK analysis was carried out in the mouse model and revealed that the bioavailability of aerosolised cetuximab was lower and elimination times were shorter in macaques than in mouse. Transgenic mice revealed the integral receptor involved in mAb distribution and PK; FcRn was expected to make a greater contribution to the recycling of cetuximab than to the transcytosis of the mAb in the airways. These results confirm the effectiveness of the inhalation route as a delivery system for lung diseases [135].

A study by Burgess et al. investigated the safety, PK, PD, and immunogenicity of VR942, an inhaled dry powder interleukin-13 mAb fragment developed for the treatment of asthma. Interleukin-13 is a core moderator of T-helper-cell-type-2 (Th-2)-driven asthma and its inhibition may enhance treatment results. This was a phase 1, randomised, double blind, placebo-controlled, ascending dose study which had the objective of reviewing the safety and tolerability of VR942 in both healthy participants and participants with asthma. They found that VR942 was well-tolerated in both cohorts and a cogent pharmacodynamic effect at 10 mg and 20 mg doses. They also showed that fractional exhaled nitric oxide (FeNO) levels, which act as a biomarker for lung inflammation, were reduced in a greater manner than seen in the placebo. These data provide evidence for the target engagement of VR942 with IL-13 pathways in airways of patients with asthma [136].

As seen in Table 2, the first-in-human phase 1/2a trial of the inhaled delivery of the SARS-CoV-2-neutralizing monoclonal antibody DZIF-10c in both healthy volunteers and SARS-CoV-2 infected individuals was carried out. The aim of the study was to evaluate the mAbs safety, PK profile, immunogenicity, and antiviral activity. A single-inhalation open label dose-escalation phase constituted as the phase 1 component of the trial. The highest tolerated dose tested will be administered to an expansion cohort of SARS-CoV-2-infected participants. Within this randomized and blinded group individuals will receive DZIF-10c or placebo both by inhalation and intravenous infusion. Although results are yet to be released, this work shows the promising progression of using inhalation to deliver CIs [137].

## 6. Future Directions

Within the last decade there have been major advances with regard to taking CIs from bench to bedside, as seen in 2010 with Sipuleucel-T (Provenge), the first CI that gained approval by the FDA. The use of living cells as a ‘drug’ allows for a significant shift from the traditional perspective of a ‘drug’ as an antibody or small molecule that provides a sole function. Conversely, cells are powerful, living agents that can conform an array of outputs, respond to environmental changes, communicate with other cells, and give rise to a series of responses that a standard drug cannot, via intricate signaling pathways [1,2]. CIs to treat cancer, also known as cancer immunotherapy, have become incredibly robust tools in the treatment of this disease. There has been a major shift in the paradigm of cancer treatments due to these cancer immunotherapies as they exhibit fewer off-target effects than chemotherapies and radiation therapies, improving anti-tumour immunity [23]. However, in recent times there has been an increase in the burden on healthcare systems due to respiratory viruses. Community viral epidemics appear to be a huge driving force in the increase of Emergency Department (ED) visits and hospitalisations, posing an increasing need to provide effective treatments for such diseases [138]. The ongoing COVID-19 pandemic continues to inflict a large public health burden, along with a strain on global finances. A fast and effective mode of action to prevent viral transmission and high mortality rates is needed. Although there are several vaccines in development and distribution, it is still difficult to predict their long-term effects, meaning alternative options must still be explored for such respiratory viral diseases. For this reason, CIs are investigated for respiratory viruses, offering huge potential for the future [139].

Aside from COVID-19, respiratory viruses continue to increase, causing numerous diseases such as acute respiratory exacerbations of chronic obstructive pulmonary disease (AECOPD), respiratory tract infections following HSCT, as well as bronchiolitis and pneumonia [32,140,141]. A large number of CIs have been tested for treating COVID-19, including ICIs, NK cell therapy, cancer vaccines, and ATC. Additionally, cell-derived therapies such as mAbs have also been heavily studied for the treatment of COVID-19 [142]. It is possible that these therapies may have a clinical application in alternative respiratory viruses moving forward. RSV in particular, is a major respiratory source of hospitalisation worldwide in infants and young children. There is a huge obstacle in designing an effective vaccine against RSV, specifically one that protects young children. In the 1960s a vaccine against RSV formulated to protect infants ended up resulting in severe RSV infection, recognised as enhanced RSV disease (ERD) [143].

Severe respiratory infection is one of the main causes of intensive care unit (ICU) admission, especially in immunocompromised patients who often have a higher likelihood of getting hypoxemic acute respiratory failure (ARF) and sepsis [144]. The COVID-19 pandemic has significantly increased the number of patients requiring intensive care. Unfortunately, ARDs has been diagnosed in approximately 40–96% of patients admitted to ICUs. The need for invasive mechanical ventilation (IMV) will differ between cases; however, one constant factor is the high mortality rates among patients requiring IMV [145]. Choosing the most appropriate and least invasive method of CI administration for patients, specifically those in the ICU, is fundamental. The inhalation route is commonly used for administering therapies for respiratory diseases such as asthma and COPD. Inhalation is advantageous as the drug is transported directly to the target organ, ensuring that drug concentration in the pulmonary tissue is high and systemic drug concentrations are low. IV administration entails the injection of the drug into the bloodstream, which is usually not the target site, meaning that the drug must travel to the target organ of the lung for respiratory diseases. Additionally, the inhalation route potentially requires a lower dose of the treatment, keeping the already very high patient costs down [113]. Nanomaterials as a drug delivery system are being explored for patients suffering with COVID-19. There is great potential in using designed nanocarriers as a more targeted therapy for treating respiratory diseases such as COVID-19. The inhalation of vaccines also has huge benefits in providing protection against bronchopulmonary infections, as well as for covering a large area when inhaled. Vaccines conjugated to NPs are considered for COVID-19 as they arm the immune system with additional defenses and can therefore increase their potency [146]. It is possible that this delivery method may be employed for CIs including cancer vaccines, ICIs, and cell-derived therapies such as mAbs [147,148,149].

## 7. Conclusions

The CI field has gained much attention in recent years and continues to expand due to promising preclinical and clinical trial results. Their application in the lung, particularly to treat respiratory viruses has yet to be fully explored. The primary objective of this review paper was to highlight the various CIs and their potential use for respiratory viruses, along with reviewing the different delivery routes for administration, such as inhaled therapies. It is evident that CIs are largely used in the treatment of cancer due to their personalised nature. However, the COVID-19 global pandemic has somewhat shifted the interest of their application towards respiratory viruses as it is a hugely relevant topic and should remain a focus in years to come.

## Figures and Tables

**Table 1:** Cellular Immunotherapy clinical trials for respiratory viruses.

NCT No	Title	Status	Company Name	Disease/Condition	Route of Administration	Intervention/Mechanism Target	Results	Phase
NCT04280224	NK cells treatment for COVID-19	Recruiting	Xinxiang medical university	Novel Coronavirus Pneumonia	Intravenous	0.1–0.2 × 10^7^ cells/kg body weight	N/A	1
NCT04365101	Natural Killer Cell (CYNK-001) Infusions in Adults With COVID-19 (CYNKCOVID)	Active, not recruiting	Celularity Incorporated	COVID-19	Intravenous	CYNK-001 infusions	N/A	1/2
NCT04457726	Part Two of Novel Adoptive Cellular Therapy With SARS-CoV-2 Specific T Cells in Patients with Severe COVID-19	Recruiting	KK Women’s and Children’s Hospital	COVID-19	Intravenous	Single infusion of SARS-CoV-2 specific T cells	N/A	1/2
NCT04386252	Phase I-II Trial of Dendritic Cell Vaccine to Prevent COVID-19 in Adults	Not yet recruiting	Aivita Biomedical, Inc	COVID-19	Intravenous	Autologous dendritic cells previously loaded ex vivo with SARS-CoV-2 spike protein	N/A	1/2
NCT04840459	Use of Monoclonal Antibodies for the Treatment of Mild to Moderate COVID-19 in Non-Hospitalised Setting	Recruiting	Sohail Rao	COVID-19	Intravenous	Single IV infusion of 700 mg bamlanivimab 10 mL of casirivimab and 10 mL of imdevimab	N/A	2
NCT04413838	Efficiency and Security of NIVOLUMAB Therapy in Obese Individuals With COVID-19 (COrona VIrus Disease) Infection (NIVISCO)	Not yet recruiting	Hospices Civils de Lyon	Obesity, COVID-19 Infection	Intravenous	IV injection within 30 min of 24 mL file containing NIVOLUMAB BMS 10 mg/mL (immune check point inhibitor targeting PD-1) on top of routine standard of care for COVID-19 infection	N/A	2
NCT04484935	Evaluate the Safety and Tolerability, for Nirsevimab in Immunocompromised Children	Recruiting	AstraZeneca	RSV infection	Intramuscular	Single fixed IM dose of nirsevimab 50 mg if body weight < 5 kg or 100 mg if body weight ≥ 5 kg, and subjects entering their second RSV season will receive a single fixed IM dose of nirsevimab 200 mg	N/A	2
NCT02325791	Study to Evaluate the Efficacy and Safety of Suptavumab (REGN2222) for Infection in Preterm infants	Completed	Regeneron Pharmaceuticals	Respiratory Syncytial Virus Infections	IM administration	Single dose of suptavumab 30 milligram per kilogram (mg/kg)	Results posted	3
NCT04268537	Immunoregulatory Therapy for 2019-nCoV	Not yet recruiting	Jianfeng Xie, Southeast University, China	COVID-19	IV administration	Anti-PD-1 antibody, 200 mg, IV, one time Thymosin, 1.6 mg sc qd, last for 5 days	N/A	2
NCT03378102	Antigen Specific Adoptive T Cell Therapy for Adenovirus Infection After Hematopoietic Stem Cell Transplantation	Recruiting	Mari Dallas, Case Comprehensive Cancer Center	Adenovirus infections occurring after allogeneic Hematopoietic Stem Cell Transplantation (HSCT).	IV administration	Subjects will receive virus-specific, antigen selected T cells within a targeted range of 1 × 10^3^–2 ×10^5^ per kg of recipient weight.	N/A	Early phase 1

**Table 2:** Inhaled cellular immunotherapies clinical trials for respiratory viruses.

NCT No	Title	Status	Company	Disease/Conditions	Route of Administration	Formulation/Device Type	Intervention/Mechanism Target	Results	Phase
NCT04631705	SARS-CoV-2-Neutralizing Monoclonal COVID-19 Antibody DZIF-10c by Inhalation	Recruiting	Florian Klein, University of Cologne	SARS-CoV-2 Infection	Inhalation	Not stated	Single dose of DZIF-10c by inhalation	N/A	1/2
NCT04822701	A Study to Test BI 767551 in People with Mild to Moderate Symptoms of COVID-19	Recruiting	Boehringer Ingelheim	COVID-19	Inhalation	Inhaler	IV injection of BI 767551 Inhalation of BI 767551	N/A	1/2
